# The Expression of Potato Expansin A3 (*StEXPA3)* and Extensin4 (*StEXT4*) Genes with Distribution of StEXPAs and HRGPs-Extensin Changes as an Effect of Cell Wall Rebuilding in Two Types of PVY^NTN^–*Solanum tuberosum* Interactions

**DOI:** 10.3390/v12010066

**Published:** 2020-01-05

**Authors:** Katarzyna Otulak-Kozieł, Edmund Kozieł, Benham E. L. Lockhart, Józef J. Bujarski

**Affiliations:** 1 Department of Botany, Institute of Biology, Warsaw University of Life Sciences—SGGW, Nowoursynowska Street 159, 02-776 Warsaw, Poland; 2 Department of Plant Pathology, University of Minnesota, St. Paul, MN 55108, USA; lockh002@umn.edu; 3 Department of Biological Sciences, Northern Illinois University, DeKalb, IL 60115, USA; jbujarski@niu.edu

**Keywords:** plant cell wall, hypersensitive response, potato virus Y-host interaction, expansin, extensin

## Abstract

The plant cell wall acts not only as a physical barrier, but also as a complex and dynamic structure that actively changes under different biotic and abiotic stress conditions. The question is, how are the different cell wall compounds modified during different interactions with exogenous stimuli such as pathogens? Plants exposed to viral pathogens respond to unfavorable conditions on multiple levels. One challenge that plants face under viral stress is the number of processes required for differential cell wall remodeling. The key players in these conditions are the cell wall genes and proteins, which can be regulated in specific ways during the interactions and have direct influences on the rebuilding of the cell wall structure. The cell wall modifications occurring in plants during viral infection remain poorly described. Therefore, this study focuses on cell wall dynamics as an effect of incompatible interactions between the potato virus Y (PVY^NTN^) and resistant potatoes (hypersensitive plant), as well as compatible (susceptible plant) interactions. Our analysis describes, for the first time, the expression of the potato expansin A3 (*StEXPA3*) and potato extensin 4 (*StEXT4*) genes in PVY^NTN^-susceptible and -resistant potato plant interactions. The results indicated a statistically significant induction of the *StEXPA3* gene during a susceptible response. By contrast, we demonstrated the predominantly gradual activation of the *StEXT4* gene during the hypersensitive response to PVY^NTN^ inoculation. Moreover, the in situ distributions of expansins (StEXPAs), which are essential cell wall-associated proteins, and the hydroxyproline-rich glycoprotein (HRGP) extensin were investigated in two types of interactions. Furthermore, cell wall loosening was accompanied by an increase in StEXPA deposition in a PVY^NTN^-susceptible potato, whereas the HRGP content dynamically increased during the hypersensitive response, when the cell wall was reinforced. Ultrastructural localization and quantification revealed that the HRGP extensin was preferably located in the apoplast, but deposition in the symplast was also observed in resistant plants. Interestingly, during the hypersensitive response, StEXPA proteins were mainly located in the symplast area, in contrast to the susceptible potato where StEXPA proteins were mainly observed in the cell wall. These findings revealed that changes in the intracellular distribution and abundance of StEXPAs and HRGPs can be differentially regulated, depending on different types of PVY^NTN^–potato plant interactions, and confirmed the involvement of apoplast and symplast activation as a defense response mechanism.

## 1. Introduction

The plant cell wall operates as a complex structure with a diversity of functions throughout the whole plant life. Through the action of many structural components, which are necessary for plant growth and acclimatization in the environment, the plant cell wall is a place of constant assembly, remodeling, or disassembly processes during the lifetime of the plant [1]. Consistent with its role in different processes, the plant cell wall structure can dynamically change, not only between different plant species, but also between different tissue types [2]. Moreover, as postulated by Tucker and Koltunow [3], a dynamic primary wall is established in young cells during division and provides flexibility and structural support. Conversely, the secondary cell wall, which is thicker and more constant, is deposited at a later stage, when the cell has stopped growing and dividing. According to Houston et al., [4], in the “battle” between plants and microbes, plants evolved a multilayer defense system, where the cell wall is the first line of defense and serves multiple purposes. The plant cell wall may act as a passive structural barrier as well as establish active induced defense [5]. Genes encoding proteins and/or enzymes capable of synthesizing or hydrolyzing components of the plant cell wall display different expressions or activities under different stress conditions, suggesting that they have roles in enabling stress tolerance through changes in the cell wall composition.

Expansins are plant cell wall remodeling proteins preliminary involved in the pH-dependent extension of the cell wall, which is referred to as acid growth [6,7]. Expansins break the hydrogen bonds between hemicellulose and cellulose microfibrils, enabling turgor-driven cell enlargement in plant growth [8]. The expansin family are involved in non-enzymatic, pH-dependent cell wall relaxation, which occurs by loosening and softening the cell wall. According to Kende et al. [9], expansins are large (225–300 amino acid residues) proteins, forming a family that can be divided into four subfamilies, namely, the α-expansins or expansin A (referred to as EXPA), β-expansins or expansin B (referred to as EXPB), and expansin-like A with expansin-like B—this classification is based on phylogenetic relationships [9,10]. An increasing amount of evidence indicates some association of expansins with environmental stress in various plants [11,12,13,14]. However, relatively little is known about controlling the functions of expansins in plant responses to biotic stress, especially in the case of viral infection. It was postulated that the lack of the expansin *A1* (*EXPA1)* gene in *Nicotiana benthamiana* induces resistance to *Turnip mosaic virus* (TuMV) [15]. Similarly, as presented by Abuqamar et al. [16], the downregulation or knockdown of *AtEXLA2* enhanced resistance to the necrotrophic fungus *Alternaria brassicicola*. Furthermore, the *EXPA4* gene negatively regulated the defense response against TMV in *N. benthamiana* [17] but enhanced abiotic stress tolerance [18]. However, aside from a role in plant abiotic stress, the influence of the *EXPA3* gene on plant–pathogen interactions was well-documented, but only for the *Arabidopsis*-cyst nematode pathosystem, by Wieczorek et al. [19]. Some functions of the expansin genes were recognized in *Solanum tuberosum* [11] but were largely restricted to genes involved in plant growth, development, and abiotic stress. The gene members of *StEXPs* showed significantly different expression levels, although *Solanum tuberosum* EXPA(StEXPA) transcripts revealed relatively high abundances in most tissues. According to Chen et al. [11], the average expression values of all *StEXPs* were 20 in the leaves, 28.5 in the roots, and only 1.3 in the potato tubers. It was postulated that the above-mentioned *EXPA3* gene in *Solanum tuberosum*, which encodes a protein of 259 amino acids (27.99 kDa) and is positioned on potato chromosome 10, was expressed in tuber sprouts, petiole, leaves, and roots, as well as the shoot apex and stamen. Additionally, it was also predicted that the upregulation of *StEXPA5*, *StEXPB3*, and *StEXPB1* increased cell wall loosening and, thus, increased the chances of infection by *Pseudomonas infestans* [11,20]. In contrast, the downregulation of *StEXPA2*, *StEXPA6*, and *StEXPA11* was shown to improve the resistance of potatoes to disease. Unlike bacterial or fungal pathogens, which are direct cell wall intruders, the functions of potato expansin genes and proteins in the plant defense against viral pathogens have rarely been demonstrated in any detail. Moreover, it is still not clear which expansins are involved in this particular type of biotic stress. However, some preliminary data regarding the involvement of *EXPA1* or *EXPA4* genes in the Solanaceous plant–virus pathosystem were reported [15,17]. We decided to focus on the role of the *StEXPA3* gene and determine the relative expression and distribution of selected kinds of expansin proteins during two types of interactions between potato virus Y (PVY^NTN^) and potato plants.

The cell wall-mediated resistance of plants against pathogens exists as three different forms of protection [21]. As postulated by Deepak et al. [21], the first form is inhibition of pathogenic cell wall-degrading enzymes, the second is structural and/or chemical remodeling of the cell wall at the point of penetration, and the third is killing of intruders. Currently, as stated by Velasquez et al. [22], structural cell wall proteins are categorized into six classes based on their composition. Four of these are extensins, i.e., proline-rich glycoproteins (PRGPs), arabinogalactan proteins (APGs), glycine-rich glycoproteins (GRGPs), and solanaceous lectins, as classes of hydroxyproline-rich glycoproteins (HRGPs) [22]. HRGP extensins play an essential role in biotic and abiotic stress responses due to their oxidative crosslinking properties. Oxidative phenolic products formed from amino acid residues within HRGPs are important factors contributing to the strengthening of the cell wall [21]. HRGPs also have a major role in pathogen arrest at the site of entry. Enhanced deposition and crosslinking of the HRGPs in suspension-cultured potato (cv. Desiree) cells contributed to the formation of a resistant barrier against pathogen infection [23]. The accumulation of HRGPs in *Nicotiana* cv. Havana cells, as induced by PVY^N^, resulted in changes to the host cell walls that provided resistance against *Erysiphe cichoracearum* [24]. In recent years, several studies were carried out to address the role of HRGP in plant–pathogen interactions and the potential involvement of HRGP in disease resistance and defense mechanisms. It was postulated by Wei and Shirsat [25] that the *AtExt1* gene regulates the *Arabidopsis–Pseudomonas syringae* interaction However, information on the participation of potato HRGPs in potato–virus interactions is still lacking. As proposed by Wycoff et al. [26], the extensin gene *HRGP4.1* plays a role in cell wall strengthening in the Fabaceae family. Therefore, we chose the *StEXT4* gene, which is similar to *EXT1* and codes HRGP proteins in *S. tuberosum*, in two types of PVY^NTN^–*S. tuberosum* interactions. Previously, we postulated that changes in the cell wall were a kind of potato defense mechanism as a response to PVY inoculation [27,28]. We demonstrated that pathogenesis-related protein (PR-2), cellulose synthase catalytic subunit CesA4, and xylan/xyloglucan deposition qwre actively changed and distributed in differing ways in the two types of interactions. Moreover, our ultrastructural analysis indicated that the cell wall composition in compatible and incompatible interactions varied, and we suggested that these were due to either cell wall reorganization/loosening or reinforcement, respectively.

Therefore, the aim of this study was to examine the expression of potato α-expansin 3 (*StEXPA3*) and extensin 4 (*StEXT4*) HRGP genes and the StEXPAs and HRGPs-extensin distribution in *Solanum tuberosum* during potato virus Y (PVY^NTN^)-susceptible and hypersensitive interactions. We demonstrated that PVY^NTN^ inoculation affected *StEXPA3* and *StEXT4* expression in both types of interactions. Moreover, the *StEXPA3* gene was induced in compatible interactions and the distribution of StEXPAs was altered. Conversely, *StEXT4* was predominantly activated in the hypersensitive response. The changes in *StEXPA3* and *StEXT4* gene expression and protein localization along with active participation in cell wall remodeling acted as a part of the defense response after PVY^NTN^ inoculation that was closely related to the type of interaction.

## 2. Materials and Methods

### 2.1. Plant Material and Virus Inoculation

Potato (*Solanum tuberosum* L.) cv. Irys (PVY^NTN^ resistance level 5.5 on a 1–9 scale) [29] and Sárpo Mira (resistance level 9) [30] were obtained from IHAR-PIB, Plant Breeding and Acclimatization Institute, Bonin Research Center. Plants were grown at conditions according to [29,31], and were inoculated mechanically, as previously described [32], with the necrotic tuber necrosis (NTN) strain of PVY at the four-leaf stage [32,33]. Potato cv. Sárpo Mira developed a hypersensitive necrotic response visible at 7 days post-inoculation [27,28]. This reaction was confirmed by the presence of the *Ny-Smira* gene on the long arm of the potato IX chromosome [34]. Potato cv. Irys reacted, with systemic necrosis visible at 10 days post-inoculation [27,28]. Both virus-inoculated and healthy control leaves were inoculated with phosphate buffer and collected 10 days post-inoculation to be tested by DAS-ELISA to check for the presence or absence of the virus [33].

### 2.2. Isolation of RNA and Genomic DNA (gDNA) for Analysis of the Expression of StEXPA3 and StEXT4 in Potato Plants with Differing Resistances Against PVY

Parallel to the localization investigations, molecular analyses were also performed. These analyses were conducted using the same time intervals and groups of mock- or PVY-inoculated potato plants of cv. Irys and cv. Sárpo Mira that were used for microscopy. For the molecular analyses, 25 mock-inoculated and 25 virus-inoculated plants were used from each cultivar. Leaf samples (0.05 g of each sample) from the buffer or virus-inoculated plants (at 0, 1, 7, 14, and 30 dpi) were collected. From each plant, we collected six leaf samples of mock-inoculated plants and six leaf samples of virus-inoculated plants at each time-point after inoculation. We repeated the whole experiment three times, using a new group of plants each time. RNA from these samples was isolated using the GeneMATRIX Universal RNA Purification Kit (EURx Sp. z o.o., Gdansk, Poland), according to the manufacturer’s protocol. From the six selected samples, RNA and gDNA were also isolated using the GeneMATRIX Universal DNA/RNA/Protein Purification Kit (EURx Sp. z o.o., Gdansk, Poland), according to the manufacturer’s protocols. The obtained gDNA was used to prepare calibration curves. The calibration curves were used to determine the efficiency of qPCR reactions for low expression transcripts. Isolated RNA was purified from gDNA by on-column DNase I digestion (EURx Sp. z o.o., Gdansk, Poland). After this, reaction samples were purified using the GeneMATRIX Universal RNA Purification Kit (EURx Sp. z o.o., Gdansk, Poland), according to the manufacturer’s protocol. RNA concentrations after purification were measured using the NanoDrop 2000 (Thermo Fisher Scientific, Waltham, MA, USA). The quality of the extracted RNA was checked using electrophoresis on a 1% agarose gel (under denaturation conditions). Additionally, a lack of RNA contamination was checked using real-time PCR with *Solanum tuberosum* elongation factor-1 alpha (*StEf1α* reference gene) [35] primers on a matrix of obtained RNA. This reaction showed that the analyzed RNA was not contaminated with gDNA. After the contamination analyses, cDNA was obtained using the NG dART RT kit (EURx Sp. z o.o., Gdansk, Poland), according to the producer’s protocol. Reverse transcription reactions were performed in a volume of 10 µL (for the reaction, we used 800 ng of RNA).

### 2.3. Real-Time Quantitative Polymerase Chain Reaction (qPCR) Expression Analysis of StEXPA3 and StEXT4 in Potato Plants with Different Resistances to PVY^NTN^

A qPCR reaction was performed using the Bio-Rad CFX96Touch^TM^ device (Bio-Rad Poland Sp. z.o.o., Warsaw, Poland) and the SsoAdvanced^TM^ Universal SYBR^®^ Green Supermix (Bio-Rad Polska Sp. z.o.o., Warsaw, Poland) with two previously prepared five-point calibration curves (based on cDNA and gDNA). The expressions of *Solanum tuberosum* expansin A3 (*StEXPA3*, GeneId: XM_006340959.2) [36,37] and *Solanum tuberosum* extensin 4 (*StEXT4*, GeneID: XM_006366812) [37,38] in both types of potato cultivars were investigated at different times after inoculation. As a reference gene, we used *Solanum tuberosum* elongation factor-1 alpha (*StEf1α*, GeneID: AB061263) in potato, as suggested by [35]. Primers for reference and investigated genes were designed using Primer3 software v. 0.4.0 (Primer3Plus, Free Software Foundation, Inc., Boston, MA, USA). The primer sequences (for all genes) and concentrations used for the reaction and corresponding annealing temperatures are presented in Table 1. The starting solution of cDNA (which was used for the preparation of the calibration curve) was a diluted 4× mix of 12 randomly selected cDNA formulations. In the case of the calibration curve based on gDNA, a 10× diluted solution of gDNA was used. In all cases, other subsequent points on calibration curves were prepared by a series of 4× dilutions. Reactions were performed in a volume of 15 µL. A total of 5 µL of 10× diluted cDNA formulation corresponding to each of the analyzed genes was added to the reaction. Conditions for the qPCR reaction are presented in Table 2.

The levels of expression of all investigated genes in the context (comparison) of the expression of the reference gene were calculated using the tool Gene Study in BioRad CFX Connect software v. 1.1 (Bio-Rad Polska Sp. z.o.o., Warsaw, Poland). Statistical analyses of the results, which included calculating the relative gene expression levels and the significant differences between the tested samples, were performed using the Gene Study tool. Results of the relative expressions of both investigated genes were normalized using *StEf1α* as a reference gene.

### 2.4. Immunofluorescence Localization and Assessment of the Quantitative Fluorescence Signal Using the Corrected Total Cell Fluorescence Method (CTCF) in Potato Plants with Different Resistances for PVY

For immunofluorescence localization, potato leaf tissue samples were fixed in 4% (*w*/*v*) paraformaldehyde in 0.1 M microtubule stabilizing buffer (MSB) of pH 6.9 containing 0.1% (*v*/*v*) Triton X-100 for 2 h at room temperature, as described by Gubler [39]. Samples were dehydrated in an ethanol series containing 10 mM dithiothreitol, embedded in butyl-methyl-methacrylate (BMM) resin, and polymerized under UV radiation for 20 h at −20 °C. Acetone was used to remove the BMM from 2 μm sections collected on silane slides (Thermo-Fischer Scientific, Warsaw, Poland) [28]. Immunofluorescence localizations of StEXPAs (EXPA3, EXPA4, EXPA6, EXPA23) and extensin (HRGP) were performed according to the procedure previously presented in [27]. Expansin localization was determined using a primary polyclonal rabbit antibody in a 1:50 dilution. The antibody was firstly projected to a C-terminal peptide of 15 amino acid residues NWQFGQTFEGKNFRV (the most immunogenic) of potato EXPA3 (UniProtId: M1AH75). Further performed bioinformatic analyses of EXPA3 C-terminal fragment revealed that the projected antibodies also detected similar C-terminal fragments located in EXPA4 (NCBI Protein ADD14632.1), EXPA6 (NCBI Protein XP_006350774.1) and EXPA23 (NCBI Protein XP_006345213.1). The primary antibody was detected using a secondary anti-rabbit IgG antibody conjugated with AlexaFluor488 in a 1:50 dilution (Jackson ImmunoResearch Europe Ltd., Cambridgeshire, UK). For localization of HRGP extensin-specific elements of the cell wall, we used a primary rat IgG monoclonal JIM11 antibody purchased from PlantProbes (Leeds, UK) and a secondary anti-rat IgG antibody conjugated with AlexaFluor488 (Jackson ImmunoResearch Europe Ltd., Cambridgeshire, UK) in a 1:100 dilution. An Olympus AX70 Provis (Olympus, Warsaw, Poland) with a UM61002 filter set equipped with an Olympus UC90 HD camera (Olympus, Poland) was used for the fluorescence imaging [28]. The fluorescence images were compared with corresponding light microscopy sections stained with crystal violet (Supplementary Figure S1). After obtaining the fluorescent images, further quantitative measurements of the fluorescence signal were performed. The quantification of the green immunofluorescence signal was determined using the ImageJ program (Version 1.52e, National Institutes of Health, Bethesda, MD, USA) within cell regions of the mock-inoculated plants and the infected Irys and Sárpo Mira plants at 0, 1, 7, 14, and 30 dpi. The levels of fluorescence signal were calculated in the form of the corrected total cell fluorescence (CTCF) at a magnification of 20× with a 1.00 zoom factor [28,40,41] using the formula previously presented in [28,42].

Estimated CTCF values were then statistically analyzed at selected time intervals for both types of reaction to PVY^NTN^ using the one-factor analysis of variance method (ANOVA). ANOVA enabled us to find the values of statistical significance when quantifying the levels of EXPAs or extensins (HGRPs). Furthermore, the mean CTCF values were evaluated at the *p* < 0.05 level of significance using post-hoc Tukey’s honest significant difference (Tukey HSD) testing in STATISTICA software (StataSoft and TIBCO Software Inc., Palo Alto, CA, USA, version 13.0).

### 2.5. Electron Microscopy Material Preparation, Immunogold Localization, and Statistical Quantification of the Cell Distribution of EXPAs and Extensins in Potato Plants with Different Resistances for PVY

Potato (infected and mock-inoculated) leaf samples (2 mm × 2 mm sections) from mock-inoculated plants and 14 and 30 dpi were fixed in 2% (*v*/*v*) paraformaldehyde and 2% (*v*/*v*) glutaraldehyde in 0.1 M cacodylate buffer (pH 7.2) for 2 h and washed four times in cacodylate buffer [27,43]. Samples were post-fixed in 2% (*v*/*v*) OsO_4_ for 2 h at 4 °C, dehydrated in an ethanol series (10%–99%) and propylene oxide, embedded in epoxy resin (EPON, Fluka, Switzerland), and polymerized at 60 °C for 24 h. Then, leaf sections (50–70 nm thick) were mounted on Formvar-coated nickel grids that were treated exactly according to the whole procedure of immunogold localization, as presented in [27]. For the localization of StEXPAs and extensins (HRGPs), the same set of primary antibodies were used as for the immunofluorescent localization (Section 2.4), including the use of the same primary antibody dilutions. To visualize the location of both proteins, we used a secondary anti-rabbit antibody (for the StEXPAs) and anti-rat antibody (for the HRGP extensins) conjugated with nanogold particles with sizes of 20 nm (anti-rabbit antibody from Sigma-Aldrich, Warsaw, Poland) and 18 nm (anti-rat from Jackson ImmunoResearch Europe Ltd., Cambridgeshire, UK), respectively, with the same dilution as for the immunofluorescent localization (Section 2.4). The labeling specificity was checked by incubating grids with material from mock-inoculated plants and by omission of the primary antibody from the incubating solution. The grids were then counterstained with 1% uranyl acetate for 5 min and washed 5 times for 2 min each with distilled water. Immunogold-labeled sections on grids were examined using transmission electron microscopy (TEM) [27]. A sample’s time-point from mock or virus inoculation for TEM observation was selected based on qPCR results and immunofluorescent localization. After examination, labeling quantification of cell wall-associated proteins was based on the method proposed by Luschin-Ebengreuth and Zechmann [44], with modifications regarding the type of statistical method and program used for statistical analyses. Data for the gold particle concentrations were investigated by analysis of variance (ANOVA) and the post-hoc Tukey HSD test in STATISTICA software (StataSoft and TIBCO Software Inc., Palo Alto, CA, USA, version 13.0). ANOVA was used as an efficient estimator of gold labeling. Each cell wall-associated protein was investigated individually. For the statistical estimation of immunogold labeling, we compared infected and healthy (mock-inoculated) plants from different cultivars at 0, 14, and 30 dpi. Gold particles in cell compartments were counted in forty 10 μm^2^ fields per image. In each combination (two mock-inoculated, cv. Irys and cv. Sárpo Mira PVY^NTN^-inoculated potato), gold particles from 200 photos were counted for StEXPAs and HRGPs.

## 3. Results

### 3.1. Expression Analysis of Expansin A3 (StEXPA3) in Compatible and Incompatible PVY^NTN^–Potato Interactions

**Figure 1:**
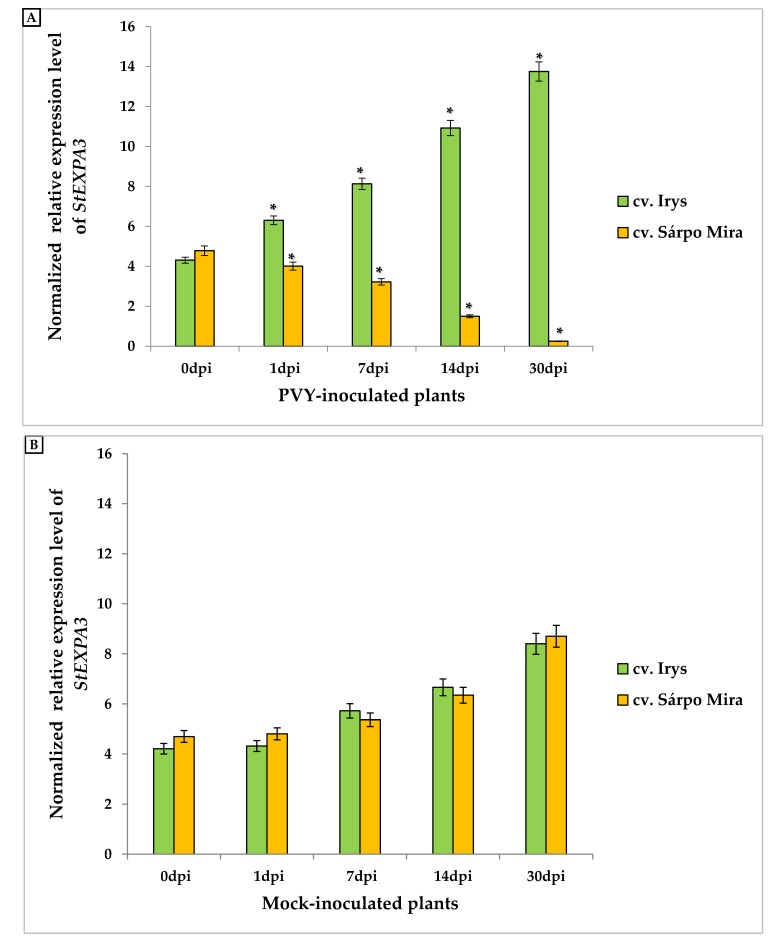
The normalized relative expression levels of *StEXPA3* calculated based on the reference gene *StEf1α* in potato virus Y (PVY)-inoculated (**A**) and in mock-inoculated (**B**) cv. Irys and cv. Sárpo Mira leaves between 0 to 30 dpi in combination with ANOVA. Mean values of normalized expression levels were evaluated at the *p* < 0.05 level of significance using a post-hoc Tukey’s honest significant difference test (Tukey HSD test). Statistically significant values on different days after mock or viral inoculation for the cultivars are marked by asterisks (*) above the mean value of the normalized expression on each bar.

We investigated and compared the expression of *StEXPA3* in two types of interactions using qPCR. Therefore, we performed analyses of expansin A3 expression in susceptible (cv. Irys) and resistant (cv. Sárpo Mira) potato cultivars between 0 and 30 days post-PVY inoculation (dpi). Additionally, mock-inoculated leaves were harvested and tested as controls to obtain a better picture of the expression patterns as a comparison between possible developmental changes and specific effects due to the virus inoculation of the potato leaves. Statistical analyses revealed that the investigated gene was expressed in virus-inoculated (Figure 1A) and mock-inoculated control leaves (Figure 1B). The normalized relative expression of *StEXPA3* was dramatically altered post-PVY inoculation in cv. Irys and cv. Sárpo Mira potato plants and was significantly different from that of mock-inoculated plants of both cultivars. The *StEXPA3* expression level was statistically significantly and strongly induced by PVY inoculation from 1 to 30 dpi in susceptible cv. Irys (Figure 1A). Moreover, a significant increase in the expression level between 1 and 30 dpi in cv. Irys was observed, estimated to be around 54.18%. Furthermore, the highest expression level was observed at 30 dpi, when severe infection symptoms and a systemic spread of PVY throughout the whole Irys potato plants occurred. In contrast, PVY-inoculated resistant Sárpo Mira plants a different change in expression. In Sárpo Mira, the normalized relative expression of *StEXPA3* steadily decreased, starting from 1 dpi in PVY-inoculated plants (Figure 1A), and was approximately 93.75% between 1 and 30 dpi of *StEXPA3*. In the mock-inoculated plants of both cultivars, a slight increase in the normalized relative expression level of *StEXPA3* was observed (Figure 1B), which was postulated to be closely related to leaf-tissue developmental processes. However, no significant differences between cultivars of the mock-inoculated plants were observed. The results indicated that *StEXPA3* was much more actively induced over time in the susceptible potato–PVY^NTN^ interactions, contrary to the hypersensitive potato–PVY^NTN^ interactions.

### 3.2. Immunofluorescence Expansins Localization and Assessment of the Quantitative Fluorescence Signal in PVY^NTN^–Potato Compatible and Incompatible Interactions

**Figure 2:**
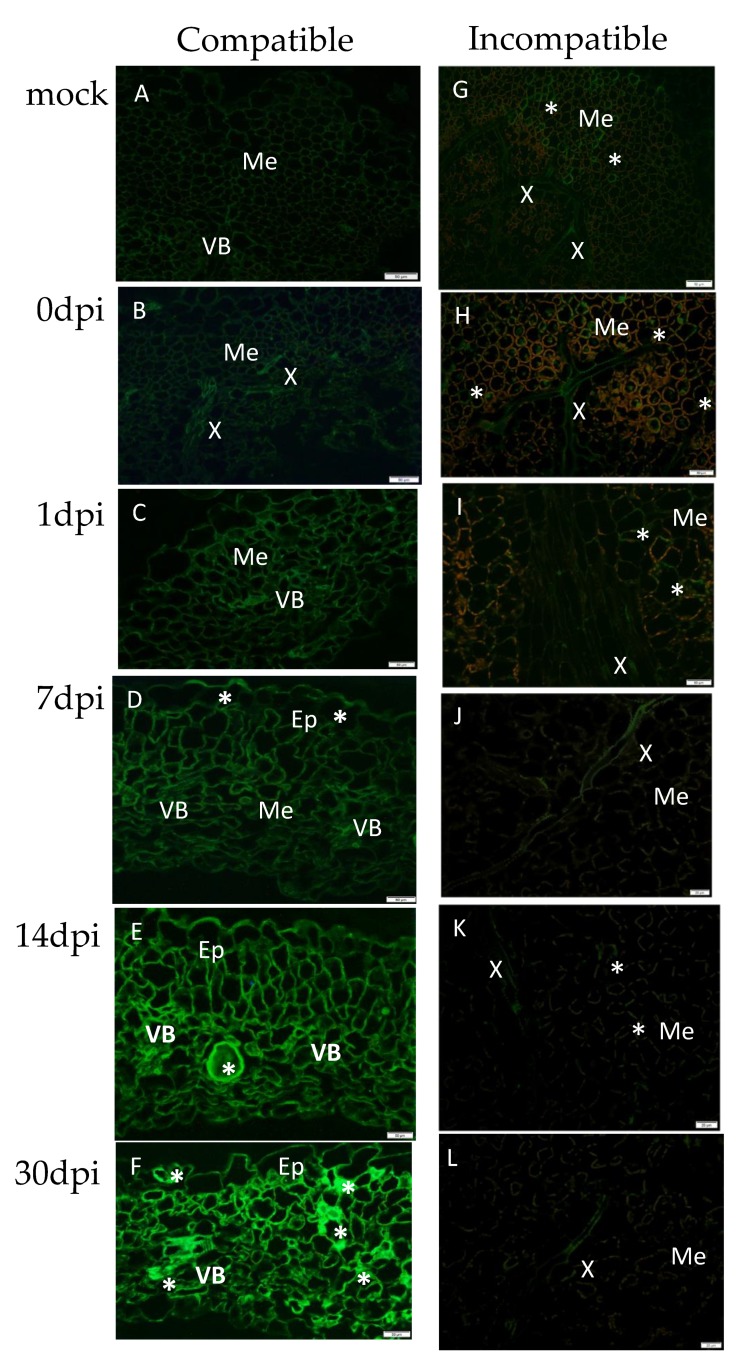
Immunofluorescence localization of *Solanum tuberosum* expansins (StEXPAs) in PVY^NTN^–potato Irys compatible (**B**–**F**) and –potato Sárpo Mira incompatible (**H**–**L**) interactions. (**A**) StEXPA green fluorescence signal in the vascular bundle and parenchyma cells in a mock-inoculated Irys potato leaf. (**B**) StEXPA green fluorescence signal in xylem tracheary elements and parenchyma cells in a PVY^NTN^-inoculated Irys potato leaf at 0 days post-inoculation (dpi). (**C**) StEXPA green fluorescence signal in the vascular bundle and mesophyll cells at 1 dpi. (**D**) StEXPAs in external cell wall of epidermis (*), mesophyll cells, and vascular tissue at 7 dpi. (**E**) StEXPAs in all leaf tissues at 14 dpi, with the most intense signal in the necrotizing mesophyll cell (*). (**F**) StEXPA signal in all leaf tissues at 30 dpi. The most intense signals (*) in the cell wall and symplast were observed in the area of necrotizing mesophyll cells, xylem tracheary elements, and stomata. (**G**) Green fluorescence signal (*) in xylem tracheary elements and mesophyll cells in a mock-inoculated Sárpo Mira potato leaf. (**H**) StEXPA green fluorescence signal (*) in xylem tracheary elements and mesophyll cells in a PVY^NTN^-inoculated Irys potato leaf at 0 days post-inoculation. (**I**) StEXPA green fluorescence signal in singular mesophyll cells at 1 dpi. (**J**) StEXPA green fluorescence signal (*) in xylem tracheary elements at 7 dpi. (**K**) Weak signals of StEXPAs in singular mesophyll cells at 14 dpi. (**L**) StEXPA green fluorescence signal (*) in xylem tracheary elements at 30 dpi. (**A**–**H**) Bar 50 µm, (**I**–**L**) bar 20 µm. Ep—epidermis; Me—mesophyll; VB—vascular bundle; X—xylem tracheary elements.

**Figure 3:**
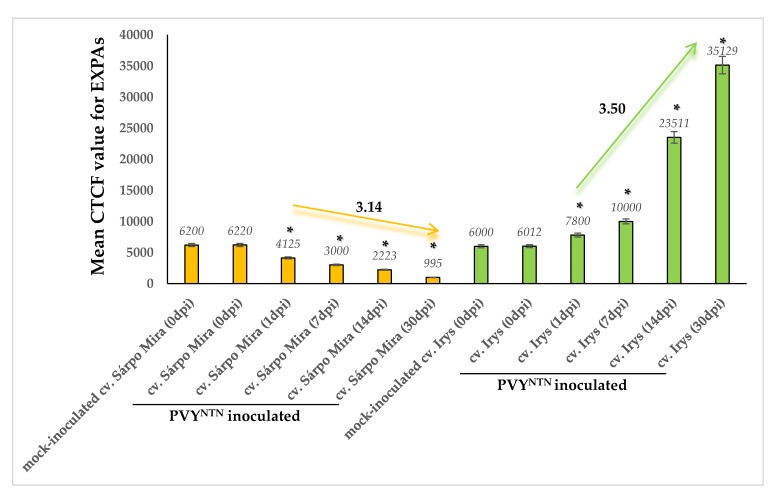
Quantitative fluorescence signals of StEXPAs using the corrected total cell fluorescence method (CTCF) in combination with ANOVA. The orange charts represent mock-inoculated and PVY-inoculated cv. Sárpo Mira (resistant) potato plants at 0, 1, 7, 14, and 30 dpi. The orange arrow represents a decrease in the fluorescent signal value between 1 and 30 dpi in virus-infected Sárpo Mira plants. The light green charts represent mock-inoculated and PVY^NTN^-inoculated cv. Irys (susceptible). The light green arrow represents an increase in the fluorescent signal value between 1 and 30 dpi in virus-infected cv. Irys plants. The mean values of CTCF were evaluated at the *p* < 0.05 level of significance using a post-hoc Tukey HSD test. Statistically significant values on different days after mock or viral inoculation for the cultivars are marked by asterisks (*) above the mean value of the CTCF on each chart bar.

The localization patterns of *Solanum tuberosum* expansins (A3, A4, A6, and A23) in the PVY^NTN^-inoculated susceptible (Irys) and resistant (Sárpo Mira) potato plant leaves were investigated by immunofluorescence analysis. The fluorescence localization of the EXPA proteins confirmed the expression pattern of the *StEXPA3* gene in both types of interactions. Fluorescence localization revealed that the expansins were induced in compatible PVY^NTN^–potato plant interactions versus incompatible or mock-inoculated plants (Figure 2 and Figure 3). In mock-inoculated plants and at zero days post-PVY inoculation, the green fluorescence corresponding to potato expansins was mainly observed in xylem tracheary elements and in singular mesophyll cells in both susceptible and hypersensitive potato plants (Figure 2A,B,G,H). Starting from one day post-PVY^NTN^ inoculation, a gradual increase in the StEXPA signal in susceptible potato plants was observed (Figure 2C–F and Figure 3). At 14 and 30 days post-PVY^NTN^ inoculation, the green fluorescence corresponding to StEXPAs was observed in all leaf tissue types. Interestingly, StEXPAs deposition was observed not only in the cell wall, but also in the symplast area, especially at 30 days post-inoculation when the intensity of the fluorescence signal was the highest. In contrast, a very weak signal was observed in the hypersensitive response almost only in the xylem tracheary element between 7 and 30 days post-PVY^NTN^ inoculation (Figure 2I–L).

An assessment of the quantitative fluorescence signal of StEXPAs using the corrected total cell fluorescence method (CTCF) strongly indicated that the strength of the fluorescent signal from the protein epitope dynamically changed after PVY inoculation in both potato cultivars. In cv. Sárpo Mira plants that were infected with PVY, we observed a dynamic and statistically significant decrease in the strength of fluorescence from 1 to 30 dpi (Figure 3). The lowest fluorescence signal was observed at 30 dpi in the PVY-inoculated plants. In contrast to the Sárpo Mira plants, changes in green fluorescence in the virus-inoculated Irys plants were quite different. After PVY^NTN^ inoculation, 3.5-fold higher fluorescence was observed in the Irys plants from 1 to 30 dpi 3.5. The strongest signal was detected in the virus-inoculated Irys plants at 30 dpi.

### 3.3. Quantitative Immunogold Localization of StEXPAs in Compatible and Incompatible PVY^NTN^–Potato Interactions

**Figure 4:**
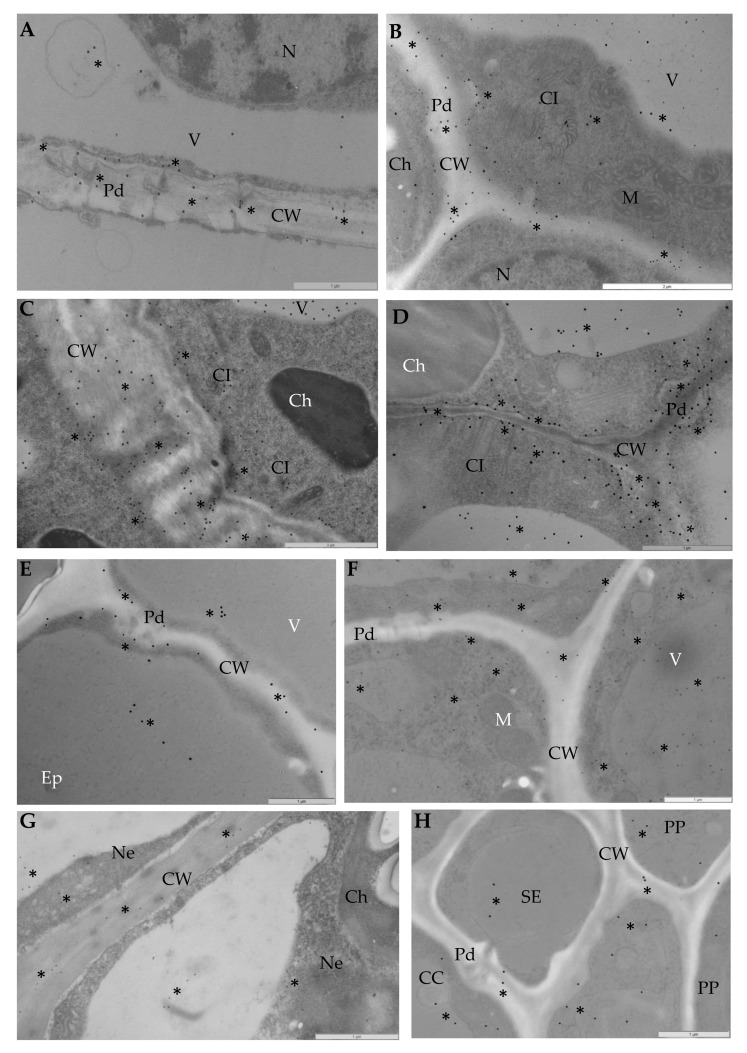
Immunogold localization of expansins in compatible (**B**–**D**) and incompatible (**F**–**H**) PVY^NTN^–potato interactions. (**A**) StEXPAs (*) in mock-inoculated mesophyll cells in the cell wall, around the plasmodesmata, in the cytoplasm, and in vacuoles; bar 1 µm. (**B**) StEXPAs (*) in the cell wall, plasmolemma, cytoplasm, and vacuoles in phloem parenchyma cells at 14 days post-virus inoculation. Virus cytoplasmic inclusion in cytoplasm; bar 2 µm. (**C**) StEXPAs (*) in the changed cell wall, plasmolemma, vacuoles, and cytoplasm of mesophyll cells at 30 days post-virus inoculation. Virus cytoplasmic inclusion in cytoplasm; bar 2 µm. (**D**) StEXPAs (*) in the cell wall, around the plasmodesmata, and along the plasmalemma of mesophyll cells at 30 days post-inoculation. Virus cytoplasmic inclusion was next to the plasmodesmata, and gold deposition was also observed in the cytoplasm and vacuoles; bar 1 µm. (**E**) StEXPAs (*) in the cell wall and around the plasmodesmata in mock-inoculated Sárpo Mira epidermis cells. Gold deposition was also observed in vacuoles; bar 1 µm. (**F**) StEXPAs (*) in the cell wall, cytoplasm, and vacuoles of phloem cells at 14 days post-virus inoculation; bar 1 µm. (**G**) StEXPAs (*) in the cell wall, cytoplasm, and vacuoles in mesophyll necrotized cells at 30 days post-virus inoculation; bar 1 µm. (**H**) StEXPAs (*) in the cell wall, cytoplasm, and vacuoles of phloem cells at 30 days post-virus inoculation; bar 1µm. CC—companion cell; Ch—chloroplast; CI—virus cytoplasmic inclusions; CW—cell wall; Ep—epidermis; M—mitochondria; N—nucleus; Ne—necrosis; Pd—plasmodesmata; V—vacuole; PP—phloem parenchyma cell; SE—sieve elements.

**Figure 5:**
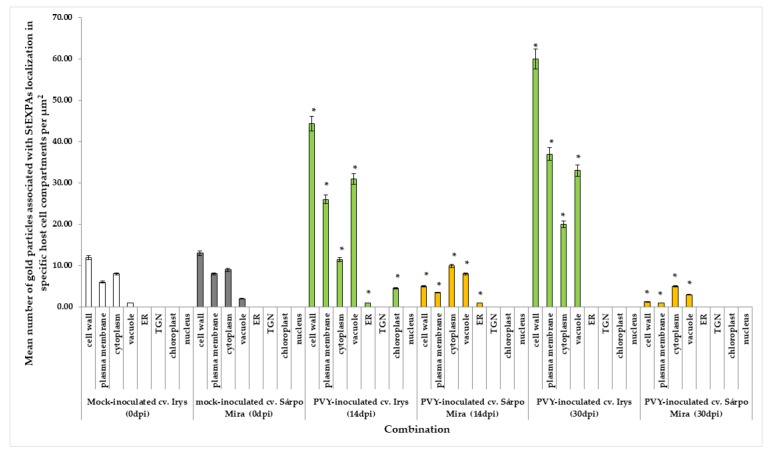
Statistical significance assessment of StEXP immunogold localization in host cell compartments of mock-inoculated and PVY^NTN^-inoculated potato plants (susceptible and resistant). The figure presents the mean numbers of gold particles located in specific compartments per µm^2^. The white charts present the StEXPAs in cell compartments of mock-inoculated cv. Irys at 0 dpi. The grey charts present the StEXPAs in cell compartments of mock-inoculated cv. Sárpo Mira at 0 dpi. The light green charts present StEXPA3 in PVY-inoculated cv. Irys at 14 dpi and 30 dpi. The orange charts present StEXPAs in PVY-inoculated cv. Sárpo Mira at 14 dpi and 30 dpi. Quantification immunogold localization was performed using ANOVA. Mean values of gold particle localization were evaluated at the *p* < 0.05 level of significance using post-hoc Tukey HSD test. Statistically significant values after mock or viral inoculation for both cultivars are marked by asterisks (*) above the bars.

Immunogold labeling was performed to precisely explore the ultrastructural localization of the StEXPA3 protein (Figure 4). Two time-points after virus inoculation were selected to compare both of the interaction types as well as the mock-inoculated potato plants to determine where the changes in StEXPAs localization were the most dynamic, based on previous fluorescence detection. StEXPAs were mainly observed in the cell wall, but also in the cytoplasm and plasma membrane in both mock-inoculated potato cultivars (Figure 4A,E). Conversely, the weakest StEXPAs deposition was in the vacuole. Quantitative ultrastructural localization clearly indicated the induction of StEXPA3 deposition 14 and 30 days after PVY^NTN^ inoculation in compatible interactions (Figure 4B–D and Figure 5). Moreover, in PVY^NTN^–Irys plant interactions, a statistically significant amount of StEXPA was detected in the cell wall, vacuole, plasma membrane, and cytoplasm. In all of these compartments, the deposition at 14 and 30 days post-PVY^NTN^ inoculation was much higher than in the mock-inoculated potato Irys. Additionally, StEXPAs were localized in loosened/expanded areas of the cell wall, as well as around plasmodesmata, especially in cells where virus particles or virus inclusion in close vicinity of the plasma membrane were observed (Figure 4B–D).

Moreover, the quantification analysis revealed statistically significant differences in StEXPA localization when comparing all of the mentioned compartments between 14 and 30 days post-PVY^NTN^ inoculation in susceptible Irys plants (Figure 5). Furthermore, the immunogold localization of StEXPA3 in hypersensitive Sárpo Mira potato plants after PVY ^NTN^ inoculation corroborated the fluorescence analysis and revealed less StEXPAs deposition compared to the mock-inoculated Sárpo Mira plants (Figure 4F–H). Interestingly, the level of StEXPA3 that was detected during the hypersensitive response was higher in the cytoplasm and vacuoles than in the cell wall. Moreover, in Sárpo Mira PVY^NTN^-inoculated plants, StEXPA localization in the cell wall was not associated with the plasmodesmata (Figure 4F,H). Furthermore, we noticed a statistically significant decrease in StEXPA localization between 14 and 30 days after virus inoculation in all compartments where StEXPAs were present.

### 3.4. Expression Analysis of Extensin 4 (StEXT4) in Compatible and Incompatible PVY^NTN^–Potato Interactions

**Figure 6:**
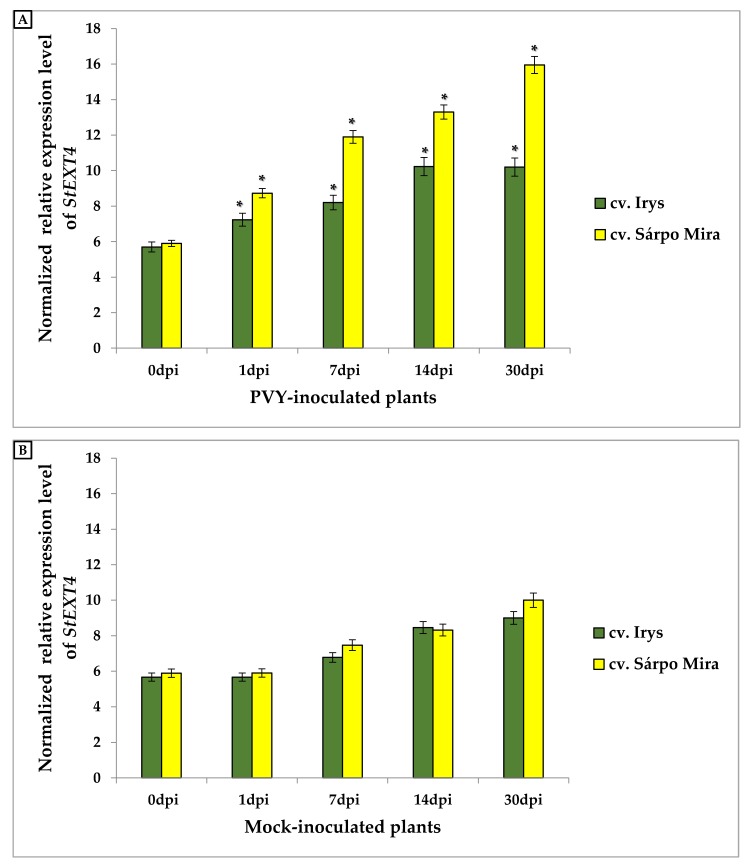
The normalized relative expression level of *StEXT4* in PVY-inoculated plants calculated based on the reference gene, *StEf1α* (**A**) and in mock-inoculated (**B**) Irys and Sárpo Mira leaves at 1, 7, 14, and 30 dpi, in combination with ANOVA. Mean values of normalized expression levels were evaluated at the *p* < 0.05 level of significance using a post-hoc Tukey HSD test. Statistically significant values on different days after mock or virus inoculation for the cultivars are marked by asterisks (*) above the mean value of the normalized expression on each bar.

Expression analyses of *StEXT4* in PVY^NTN^-inoculated and mock-inoculated Irys and Sárpo Mira potato plants were performed at time intervals corresponding to those employed in the *StEXPA3* analyses. Statistically significant changes in the normalized relative expression levels of *StEXT4* in PVY^NTN^-inoculated (Figure 6A) and mock-inoculated (Figure 6B) potato plants were observed. The *StEXT4* expression levels were induced in a statistically significant manner in Irys plants between 1 and 14 days post-virus inoculation. At 30 dpi, the *StEXT4* expression levels in PVY-infected Irys plants did not significantly differ from those at 14 dpi. Conversely, PVY^NTN^-inoculated Sárpo Mira plants exhibited an increase in expression from 1 to 30 dpi (Figure 6A). Moreover, the induction of *StEXT4* expression in PVY-inoculated Sárpo Mira plants was much higher than in PVY-inoculated susceptible Irys (Figure 6A). Induction of the expression of *StEXT4* between 1 and 30 days after PVY^NTN^ inoculation of Irys plants was 29.12%. Conversely, in virus-inoculated Sárpo Mira, the expression of *StEXT4* increased by over 45% in the corresponding time interval. Furthermore, in mock-inoculated potato plants, the expression of *StEXT4* slightly increased, but was not considered to be statistically significant (Figure 6B). The results clearly indicated that *StEXT4* was much more actively induced in the hypersensitive response to PVY^NTN^ inoculation than in susceptible potato plants.

### 3.5. Immunofluorescence Localization of Hydroxyproline-Rich Glycoprotein (HRGP) Extensins with the Assessment of the Quantitative Fluorescence Signal in Compatible and Incompatible PVY^NTN^–Potato Interactions

**Figure 7:**
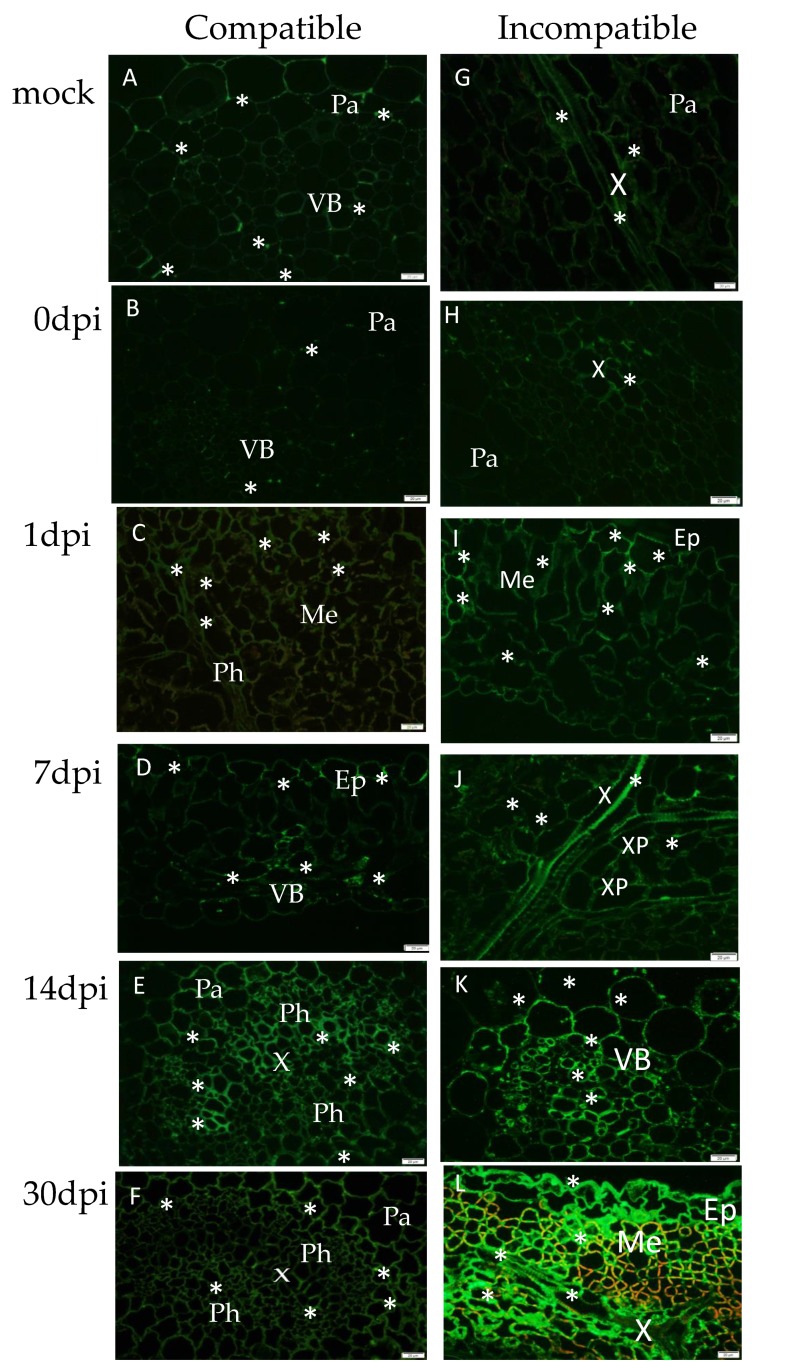
Immunofluorescence localization of hydroxyproline-rich glycoprotein (HRGP) extensins in compatible (**B**–**F**) and incompatible (**H**–**L**) PVY^NTN^ interactions. (**A**) HRGP green fluorescence signal (*) in xylem tracheary elements and parenchyma cells in mock-inoculated Irys potato plants. (**B**) HRGP weak green fluorescence signal in parenchyma cells around the vascular tissue at 0 dpi. (**C**) HRGPs in phloem and mesophyll cells at 1 dpi. (**D**) Green fluorescence signal in the epidermis and the vascular bundle at 7 days post-virus inoculation. (**E**) HRGPs in the vascular bundle and parenchyma cells around the vascular tissue at 14 dpi. (**F**) HRGPs in the vascular bundle and parenchyma at 30 days post-virus inoculation. (**G**) HRGP green fluorescence signal (*) in xylem tracheary elements in mock-inoculated Sárpo Mira potato plants. (**H**) HRGP green fluorescence signal in xylem tracheary elements in Sárpo Mira potato plants at 0 dpi. (**I**) HRGPs in the mesophyll and the epidermis at 1 dpi. (**J**) HRGPs in xylem tracheary elements and xylem parenchyma cells at 7 dpi. (**K**) HRGPs in the vascular bundle at 14 dpi. (**L**) HRGPs in the epidermis, mesophyll, and xylem tracheary elements at 30 days post-virus inoculation; bar 20 µm. Ep—epidermis; Me—mesophyll; Pa—parenchyma; Ph—phloem; VB—vascular bundle; X—xylem tracheary elements; XP—xylem parenchyma.

**Figure 8:**
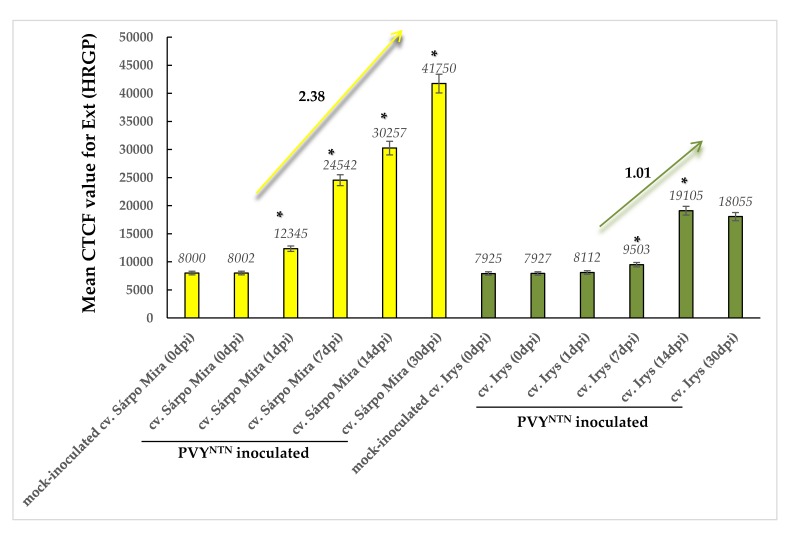
The quantitative fluorescence signal of extensins (HGRPs) was performed using the corrected total cell fluorescence method (CTCF) in combination with ANOVA. The yellow charts represent mock-inoculated and PVY-inoculated Sárpo Mira (resistant) potato plants at 0, 1, 7, 14, and 30 dpi. The yellow arrow represents an increase in the fluorescent signal value between 1 and 30 dpi in virus-infected Sárpo Mira plants. The dark green charts represent mock- and PVY^NTN^-inoculated Irys (susceptible). The dark green arrow represents an increase in the fluorescent signal value between 1 and 14 dpi in virus-infected Irys plants. Mean values of CTCF were evaluated at the *p* < 0.05 level of significance using the post-hoc Tukey HSD test. Statistically significant values after mock or virus inoculation are marked by asterisks (*) above the mean value of the CTCF on each bar.

Immunofluorescence of the hydroxyproline-rich glycoprotein group of extensins was performed in PVY^NTN^-susceptible Irys and resistant Sárpo Mira potato plant interactions. In the mock-inoculated plants and those between zero and one day after virus inoculation of susceptible Irys plants, the fluorescence HRGP signal displayed similar intensity levels. Conversely, at 7 and 14 days after virus inoculation, a visible induction of HRGPs was observed (Figure 7A–F). The HRGP signal was mainly detected in the vascular bundle and parenchyma or mesophyll cells around the vasculature of susceptible potato plants (Figure 7D–F). In contrast to the compatible interaction type, the HRGP signal gradually increased in the hypersensitive response reaction after PVY^NTN^ inoculation (Figure 7I–L and Figure 8) compared with the mock-inoculated Sárpo Mira potato plants (Figure 7G) and susceptible Irys plants (Figure 7B–F and Figure 8). Moreover, HRGP deposition was observed in xylem tracheary elements with singular mesophyll cells, from one to seven days after PVY inoculation (Figure 7I–J). Conversely, at 30 days post-PVY^NTN^ inoculation, the green fluorescence signal of HRGP was observed in all leaf tissue types. Moreover, the signal was detected not only in cell walls, but also in the symplast of vascular tissues and mesophyll cells (Figure 7L).

Statistical analyses of CTCF strongly indicated that the strength of the fluorescent signal from HGRPs was increased in PVY-inoculated potato plants in both cultivars (Figure 8). However, temporal and dynamic extensin fluorescent signal changes were different in Irys and Sárpo Mira potato plants. Induction of the fluorescent signal was observed from 1 to 14 days post-PVY^NTN^ Irys plant inoculation and was 1.01-fold higher, especially between 7 and 14 days post-inoculation (Figure 8). Thirty days post-virus inoculation, the fluorescent signal was slightly lower than at 14 dpi, but this was not statistically significant. The dynamics of the fluorescent signal induction from extensins was much greater in the virus-inoculated Sárpo Mira plants, where the signal was 2.38-fold higher. Moreover, the highest fluorescent signal in PVY-inoculated Sárpo Mira plants was observed at 30 dpi. Furthermore, the HRGP signal was higher in the virus-inoculated Sárpo Mira plants than at the highest level of HRGP detection in the Irys plants.

### 3.6. Immunogold Localization of Extensins (HGRPs) with Quantification in Compatible and Incompatible PVY^NTN^–Potato Interactions

**Figure 9:**
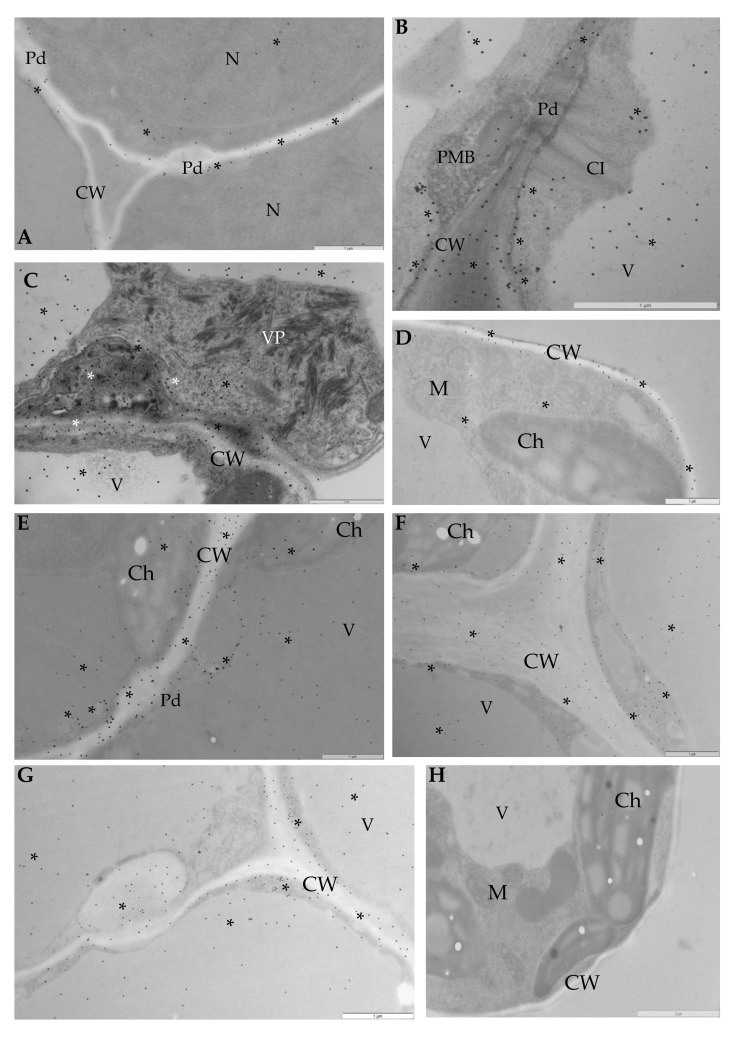
Immunogold localization of HGRP extensins in compatible (**B**–**C**) and incompatible (**E**–**G**) PVY^NTN^–potato interactions. (**A**) HRGPs (*) in mock-inoculated phloem cells in the cell wall, cytoplasm, and nucleus; bar 1 µm (**B**) HRGPs (*) in the cell wall, plasmolemma, cytoplasm, and vacuoles in mesophyll cells at 14 days post-virus inoculation. Virus cytoplasmic inclusion next to plasmodesmata; bar 1 µm. (**C**) HRGPs (*) in the changed cell wall, plasmolemma, vacuoles, and cytoplasm mesophyll cells at 30 days post-virus inoculation. Virus particles in cytoplasm; bar 1 µm. (**D**) HRGPs (*) in the cell wall and cytoplasm of mesophyll cells of mock-inoculated Sárpo Mira plants; bar 1 µm. (**E**) HRGPs (*) in the cell wall and around the plasmodesmata. Gold deposition in chloroplasts and vacuoles, along the plasmolemma, and in the membranous vesicular structure in mesophyll cells at 14 days post-virus inoculation; bar 1 µm. (**F**) HRGPs (*) in the cell wall and plasmolemma in phloem parenchyma cells. Gold deposition in cytoplasm, vacuoles, and chloroplasts at 30 days post-virus inoculation; bar 1 µm. (**G**) HRGPs (*) in the cell wall and plasmolemma in xylem parenchyma cells and xylem tracheary elements at 30 days post-virus inoculation. Gold deposition in cytoplasm and vacuoles; bar 1 µm. (**H**) Lack of deposition in Sárpo Mira mesophyll cells at 14 days post-inoculation. Section incubated with pre-immune serum; bar 2 µm. Ch—chloroplast; CI—virus cytoplasmic inclusions; CW—cell wall; M—mitochondria; N— nucleus; Pd—plasmodesmata; PMB—paramular bodies; V—vacuole; VP—virus particles; X—xylem tracheary elements; XP—xylem parenchyma.

**Figure 10:**
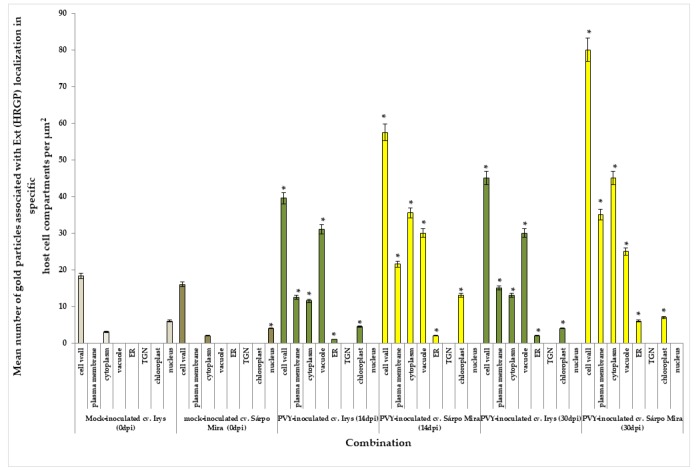
Statistical significance assessment of immunogold localization of extensins (HGRPs) in host cell compartments of mock- and PVY-inoculated potato plants (susceptible and resistant). The figure presents the mean numbers of gold particles located in specific compartments per µm^2^. The light bronze charts present HRGP extensin immunogold localization in cell compartments of mock-inoculated Irys at 0 dpi. The dark bronze charts present HRGP extensin immunogold localization in cell compartments of mock-inoculated Sárpo Mira at 0 dpi. The dark green charts present HRGP extensin immunogold localization in PVY-inoculated Irys at 14 and 30 dpi. The yellow charts present HRGP extensin epitope immunogold localization in PVY-inoculated Sárpo Mira at 14 and 30 dpi. Quantification of immunogold localization was performed using ANOVA. The mean values of gold particles localization were evaluated at the *p* < 0.05 level of significance using the post-hoc Tukey HSD test. Statistically significant values after mock or virus inoculation are marked by asterisks (*) above the bars.

For the HRGP immunogold labeling analysis, the two time-points of 14 and 30 days post-PVY^NTN^ inoculation were chosen for the two interaction types based on the fluorescence detection analysis, which indicated that these time-points corresponded to the occurrence of the most dynamic extensin deposition changes. Gold particle localization indicated that HRGPs were induced after PVY^NTN^ inoculation (Figure 9 and Figure 10) compared with the mock-inoculated Irys and Sárpo Mira plants. Moreover, in the mock-inoculated potato plants, HRGP localization was detected almost only in the cell wall (Figure 9A,D), whereas HRGPs were also observed in the symplast (cytoplasm, plasma membrane, vacuoles, or even chloroplasts) in PVY^NTN^-inoculated potato plants (Figure 9B,C,E–G).

Furthermore, the quantification of immunogold labeling clearly indicated that the induction of extensin deposition was much higher in the hypersensitive response of the Sárpo Mira plants than in the Irys plants (Figure 10).

Additionally, the localization was most intense in the cell wall of the Sárpo Mira plants, but HRGP induction was also observed in the symplast, especially in the cytoplasm, plasma membrane, and vacuoles. Moreover, HRGP accumulation was the most intense in the cell wall and cytoplasm, and the least intense in the plasma membrane 14 and 30 days after PVY^NTN^ inoculation during the hypersensitive response. In contrast, HRGPs mainly accumulated in the cell wall and vacuoles in the Irys plants, suggesting that different HRGP distributions occurred as parts of different reaction types in response to virus inoculation. Regardless of the type of interaction, for both StEXPAs and HRGPs, a lack of gold deposition was observed in sections that were incubated with pre-immune serum as a negative control for localization (Figure 9H).

## 4. Discussion

### 4.1. StEXPA3 Gene Expression and StEXPAs Localization in PVY^NTN^–Potato Interactions

The plant cell wall is the first contact point during biotic stress and plays an important role in the activation and regulation of defense response strategies. Many publications proposed models of plant cell wall modifications in response to pathogens such as nematodes, bacteria, or fungi, which directly break down the cell wall [45,46]. However, there is still limited information regarding cell wall remodeling as part of the plant defense response against viral pathogens. Our previous studies precisely described the ultrastructural changes resulting from PVY^NTN^–potato compatible and incompatible interactions. Moreover, our findings revealed that the active distribution of cell wall proteins, such as pathogenesis-related protein 2 (PR-2), catalytic subunit cellulose synthase A4 (CesA4), and xyloglucan metabolism, is associated with the type of PVY interaction [27,28]. Based on the localization of cell wall components, our data suggested that cell wall reinforcement occurred in the hypersensitive response, whereas in susceptible PVY–potato interactions, the rearrangement of apoplast could be regarded as a cell wall loosening process. Therefore, in this study, we examined the influence of PVY^NTN^ inoculation on the gene expression as well as the localization and abundance of the two main proteins that participated in the strengthening and loosening the cell wall, i.e., extensin HRGPs and *Solanum tuberosum* expansins, respectively. The cell wall dynamics determine the cell shape as well as its functions in development process responses to environmental factors, in addition to contributing to the strength and integrity of the cell [2]. The cell wall also has crucial functions in various activities that provide both mechanical strength and plasticity, which are necessary properties for the development of tissues and plant organs [1]. Plant growth requires the modulation of cell shape and size; therefore, active changes and the regulation of expansins have important influences on cell wall plasticity [47,48]. According to Pien et al., [49] expansins participate in the leaf initiation process. Moreover, the overexpression or suppression of expansin genes affect leaf development. Cho and Cosgrove [50] postulated that the suppression or overexpression of *AtEXP10,* respectively, resulted in either a reduction in leaf size or longer leaves. The authors stated that plants developed larger leaves if *AtEXPA10* and *PnEXPA1* were overexpressed, indicating that they are important for leaf initiation and growth. Furthermore, it was postulated that *AtEXP10* and *NtEXPA5* function in the control of leaf size through their actions on cell wall rheology [51,52]. Our analysis determining the expression of *StEXPA3* in potato plants between 1 and 30 days after mock inoculation revealed that the level of *StEXPA3* in potatoes slightly increased during leaf development. It was concluded that the level of *StEXPA3* expression is related to plant growth. Additionally, Sampedro and Cosgrove [53] stated that expansins may disrupt microtubular array, cellulose deposition, and cell wall thickening, which are all required for the development of stomatal guard cells, resulting in altered leaf morphology. Thirty-six expansin genes were identified in potato plants, as well as their corresponding genes from the genomes and transcriptomes [11]. It was postulated that *StEXP* genes have a potential role in potato growth as well as in abiotic and biotic stress tolerance. Moreover, these genes provide fundamental resources for future studies regarding potato breeding. Interesting analyses were revealed by Ding et al. [20], who found that *Solanum tuberosum* expansin genes loosened cell walls, thereby leading to vulnerable cells that were easily damaged by biotic invaders. It was postulated that the upregulation of *StEXPA5* and *StEXPB3* increases wall loosening, thus increasing the chance of *Phytophthora infestans* infection. The downregulation of *StEXPA2*, *StEXPA6*, *StEXPA11, StEXPA15*, and other β-class expansins, as shown by Chen et al. [11], was able to improve the resistance of potato to disease triggered by *P. infestans*. However, limited studies exist regarding the function of expansins in the plant response to biotic stress pathogens. Moreover, information about the role of expansin genes and proteins in response to plant viruses has rarely been reported. Based on the findings concerning other pathogens and taking into account our previous studies on ultrastructural changes and the localization of structural and remodeling cell wall proteins, we demonstrated for the first time that in compatible interactions (susceptible potato), the *StEXPA3* gene and selected StEXPAs protein were induced as effects of PVY^NTN^ inoculation, whereas in incompatible interactions (hypersensitive potato), *StEXPA3* gene expression and StEXPA proteins decreased between 1 and 30 days after virus inoculation. Our findings were consistent with the conclusions based on other studies of other plant–pathogen interactions, in which lower levels of expansins resulted in more resistant plant hosts, similar to the findings concerning another expansin, EXPA1, in *Nicotiana benthamiana*, as postulated by Park et al. [15], who showed that silencing *NbEXPA1* inhibited tobacco growth and infection by *Turnip mosaic virus* (TuMV, *Potyvirus*). Furthermore, the overexpression of *NbEXPA1* promoted not only virus infection, but also its cell-to-cell transport. Marowa et al. [1] found that a low level of expansins may be regarded as part of a plant’s defense response. In contrast to these findings, previous studies presented by Yang et al. [54] demonstrated that the expression tendencies of *Arabidopsis* genes related to the cell wall, such as (pectin methylesterase 3) *PME3*, *(xyloglucan transferase 6) XTH6*, and *EXP10*, depended on the area of harvested probes, and the accumulation of mRNA transcripts was reduced after TuMV infection. It was also emphasized that sRNAs corresponding to EXP10, PME3, and XTH6 were induced in response to TuMV at 10 days post-inoculation. Additionally, our presented results were similar to investigations conducted by Chen et al. [17], who stated that low levels of EXPA4 in *Nicotiana benthamiana* plants significantly downregulated the sensitivity of tobacco to infection by the constructed *Tobacco mosaic virus*–green fluorescence protein (TMV–GFP). Moreover, Chen et al. [17] postulated that *EXPA4* overexpression accelerated virus reproduction and disease development in tobacco. In our presented studies, we demonstrated the localization of StEXPAs in PVY^NTN^–potato interactions for the first time. The *StEXPA3* gene is located on chromosome 10, and different *StEXP* genes were found to be generally differentially expressed according to tissue [11]. Moreover, the expression profiling proposed by Chen and colleagues [11] indicated that *StEXPA3* was expressed in roots, tuber sprouts, the shoot apex, petiole, and leaves. We demonstrated that the StEXPA signal gradually increased in compatible PVY–potato interactions, while gradually decreasing during the hypersensitive response. Moreover, the StEXPA proteins were predominantly observed between one and seven dpi in vascular tissues and between 14 and 30 dpi in almost all leaf tissues in compatible interactions in cell walls. They were also observed in the cell symplast, with the epidermis, stomata, and vascular bundles taken into special consideration. Conversely, in the hypersensitive response, a weak signal was detected in xylem elements. Interestingly, the quantitative ultrastructural localization clearly demonstrated that the StEXPAs proteins was preferably deposited in the symplast area (plasma membrane, cytoplasm) in the hypersensitive, contrary to susceptible plants, in which StEXPAs were mainly localized to the cell wall. These findings were compatible with the assumption that the distribution of expansins was significantly different and related to the type of PVY^NTN^–potato interaction. Furthermore, we demonstrated that the StEXPAs were localized in the plasmodesmata area in compatible interactions and also in close vicinity to cytoplasmic inclusions—typical for *Potyvirus*—which was contrary to what was observed for the hypersensitive response. This result was similar to the findings presented by Park et al. [15], who postulated that NbEXPA1 was a plasmodesmata-localized protein. Further studies are necessary to investigate the effects of overexpression and/or silencing of the *StEXPA3* gene on potato infection with PVY and, moreover, localization with antibodies precisely directed to the conserved sequences of specific potato expansins in the context of their roles in the local transport of viruses and potential direct cooperation with virus proteins.

### 4.2. StEXT4 Gene Expression and Hydroxyproline-Rich Glycoprotein Extensins (HRGPs) Localization in PVY^NTN^–Potato Interactions

The plant cell wall is known as a “mediator” of resistance in the defense response against different pathogens. When a plant is exposed to biotic stress, both the composition and structure of the plant cell wall can be modified, which involves different groups of extensins. The HRGP superfamily present in plant cell walls is known to be involved in host–pathogen interactions [55,56]. However, the specific functions of individual HRGPs in the plant defense response to different pathogen groups are still poorly described. In our previous studies, we demonstrated that HRGP localization was more intense 10 days after virus inoculation in both susceptible and resistant plants compared to control plants. Based on the present analyses, we postulate that the expression of the *StEXT4* gene, which is a *Solanum tuberosum* HRGP gene, was induced in a statistically significantly manner in PVY^NTN^-inoculated leaf tissues between 1 and 30 dpi in both the susceptible and hypersensitive potato responses. Moreover, the presented results demonstrated that the gradual induction of *StEXT4* expression was more rapid and intense in the hypersensitive Sárpo Mira plants than in the susceptible potato Irys and mock-inoculated potato plants. Contrary to our results, Shimizu et al. [57], based on a microarray analysis of cell wall-related gene expression in response to *Rice dwarf virus infection*, postulated that two other extensins—proline-rich glycoproteins and glycine-rich glycoproteins—were markedly suppressed. Additionally, our results were partially consistent with the findings presented by Zheng et al. [58], who underlined that glycine-rich cell wall extension genes were markedly upregulated in a disease-resistant rice cultivar, but were downregulated or unchanged in susceptible cultivar infection by rice stripe virus based on a probe analysis two days after inoculation. On the other hand, our investigations were complementary to the findings of Benhamou et al. [59], who stated that TMV infection, as a biotic factor, induced HRGP in tobacco. This was similar to the analyses presented by Wycoff et al. [26], who demonstrated that transgenic tobacco plants expressing the GUS (β-glucuronidase)-HRGP 4.1 gene that were inoculated with TMV had increased levels of GUS activity in comparison to control plants. Furthermore, our *StEXT4* gene expression results seemed to be consistent with the conclusions presented by Chakraborty and Basak [60], who revealed that in the relative expression of HRGP during mungbean yellow mosaic India virus (MYMIV) in *Vigna mungo* (*Fabaceae*), two types of interaction were induced between 1 and 30 dpi. Additionally, the expression of HRGP in incompatible interactions (resistant plants) significantly and gradually became more intense than in a susceptible cultivar during a compatible interaction. Interestingly, abundances of the other groups of extensin genes—proline-rich glycoproteins and glycine-rich glycoproteins—increased significantly throughout the study time in resistant cultivars, whereas susceptible cultivars exhibited significant changes in gene expression [60]. The data presented by Chakraborty and Basak clearly indicated that different types of extensin groups could be regulated in different ways for different plant–virus interactions.

Induced deposition of HRGPs in PVY^NTN^–potato interactions was mainly noticed in vascular tissues starting from one day after virus inoculation. Conversely, at 30 days after inoculation, the HRGP signal was observed in almost all leaf tissues, especially in hypersensitive Sárpo Mira plants. In *Fusarium oxysporum*–wax guard plant interactions, a similarly higher level of extensin deposition was observed in resistant cultivars in an analysis conducted by Xie et al. [61]. The authors explained that HRGPs in the cell wall were involved in inhibiting the progress of pathogens in xylem tracheary elements. The idea that HRGPs preferentially accumulate in cultivars resistant to pathogens was also suggested by Basavaraju et al. [62]. Our ultrastructural localization of HRGPs demonstrated that these types of extensins were preferentially localized in the cell wall and apoplast, but their deposition in the symplast was also documented. Quantitative analysis revealed that the symplast location of HRGPs was more abundant in hypersensitive interactions and were found in the cytoplasm, plasma membrane, and vacuoles. Similar analyses were presented by O’Connel et al. [63], who demonstrated a high concentration of HGRPs not only in the cell wall, but also in membranous structures and close to the plasma membrane during a study of resistance to *Pseudomonas syringae* and *Colletotrichum* fungi. The presence of HRGPs in plasmalemma as well as in intracellular organelle membranes was previously noticed by Jeffree and Yeoman [64]. Furthermore, the location of HRGPs in vesicles and plasma membranes was confirmed by Jose-Estamyol and Puigdomenech [65]. A similar conclusion was drawn based on studies showing that HRGPs preferentially accumulated in resistant plants compared to susceptible plants. The symplastic deposition of HRGPs and other extensins explains their synthesis and maturation process. The mRNA of the extensins binds to the ribosomes and the nascent polypeptide binds to the signal recognition particle(s) and move to the endoplasmic reticulum (ER), where the signal recognition particle binds to its receptor. Subsequently, the signal peptide is inserted to the endoplasmic reticulum, and enters the secretory pathway [66], moving to the Golgi network. The extensin is glycosylated, with O-linked arabinosylation beginning in the cis-Golgi cisternae [67]. Moreover, double-immunolabeling experiments with colloidal gold showed that extensin moves through the entire Golgi pathway, being processed like xyloglucan [67]. Eventually, the extracellular matrix proteins end up in the lumen of a secretory vesicle. It should be emphasized that JIM11 monoclonal antibodies could serve to detect the plant defence response, as previously proposed for the *Musa*–*Fusarium oxysporum* pathosystem [68], thereby recognizing the hydroxyproline-rich glycoproteins belonging to the4 extensin group, but also including some lectins in the members of the potato family (*Solanaceae*) [69]. Cell wall extensins can be converted into soluble glycoproteins using prolyl hydroxylase and oxyprolyl arabinosyl transferase. Both enzymes are localized in the Golgi apparatus, and the lectins in the *Solanaceae* plants are localized both intracellularly and extracellularly. The accumulation of lectins was observed after inoculating TMV into hypersensitive varieties of *Nicotiana tabacum* and *Datura metel* [70,71,72]. In plants with systemic necrosis or systemic infection of susceptible varieties by *Cucumber mosaic virus* (CMV), no such enhancement was observed. Tobacco vacuolar protein was induced by TMV in the hypersensitive variety, as well as by wounding [73]. Increased activity in the readily soluble lectin protein fraction was registered approximately the next day after inoculation of tobacco and wild type potato plants with viruses, inducing a hypersensitive reaction. However, no increase was observed upon inoculation of the pathogen causing systemic infection in susceptible plants [72,74].

Therefore, the analysis of the HRGP extensin gene *StEXT4*, as well as HRGP localization, provide information demonstrating that these extensin groups are involved in PVY^NTN^–potato interactions, but that the cell wall accumulation in resistant Sárpo Mira plants was more intense. Moreover, HRGP extensins may participate in the reinforcement of the cell wall during a hypersensitive reaction. HRGP may be regarded as a key component of the cell wall contributing to pathogen resistance. However, changes in the intra- and extracellular distribution and relative abundance of extensin components can be differently regulated, depending on the different types of interactions. Further molecular and cellular studies regarding different extensin groups in PVY^NTN^–potato interactions are needed to define the precise functions of all extensins and their contributions to resistance mechanisms.

## Figures and Tables

**Table 1:** Accessions of genes, sequences of primers, annealing temperatures, concentrations, and product lengths for the real-time qPCR.

Genes	*Gene ID*	Forward Primer	Reverse Primer	Temperature of Primer Annealing (°C)	Concentration in Reaction (μM)	Product Length (bp)
**Investigated**						
*StEXPA3*	XM_006340959.2	5′-TGCCGTCAATGCCAGAATCC-3′	3′-CACCGTAGAACGTAGCGTGG-5′	58	0.5	74
*StEXT4*	XM_006366812.2	5′-GATAAGCCATTAGACGCCATT-3′	3′-TCGCCAGAACTATCACAGAA-5′	58	0.5	100
**Reference**						
*EF1a*	AB061263	5′-GGTGATGCTGGTATGGTTAAG-3′	3′-GGTCCTTCTTGTCAACATTCTT-5′	58	0.5	148

**Table 2:** Conditions of the qPCR reaction for the investigated genes, *StEXPA3* and *StEXT4*, and the reference. Fluorescence signal reading set on the end of the stage is marked by an asterisk (*).

Program	Parameters
Preliminary denaturation	95 °C for 5 min
Amplification (35 cycles)	95 °C for10 s 58 °C for 10 s 72 °C for 20 s *
Melting curve	65–95 °C; 0.5 °C/s

## Supplementary Materials

The following are available online at https://www.mdpi.com/1999-4915/12/1/66/s1, **Figure S1:** Immunofluorescence localizations with corresponding sections from light microscopy analyses. **S1A** Immunofluorescence localization of StEXPAs in PVY^NTN^-potato Irys, **S1B** Immunofluorescence localization of StEXPAs in PVY^NTN^-potato Sárpo Mira, **S1C** Immunofluorescence localization of HRGPs-extensins in compatible PVY^NTN^-interaction, **S1D** Immunofluorescence localization of HRGPs-extensins in incompatible PVY^NTN^- interaction.
