# Supplementary material for: The Expression of Potato Expansin A3 (StEXPA3) and Extensin4 (StEXT4) Genes with Distribution of StEXPAs and HRGPs-Extensin Changes as an Effect of Cell Wall Rebuilding in Two Types of PVYNTN–Solanum tuberosum Interactions
Source: Viruses. 2020 Jan 5;12(1):66. doi: 10.3390/v12010066 (PMC7020060; doi:10.3390/v12010066)
Supplement: Supplementary file 1 [file viruses-12-00066-s001.pdf]

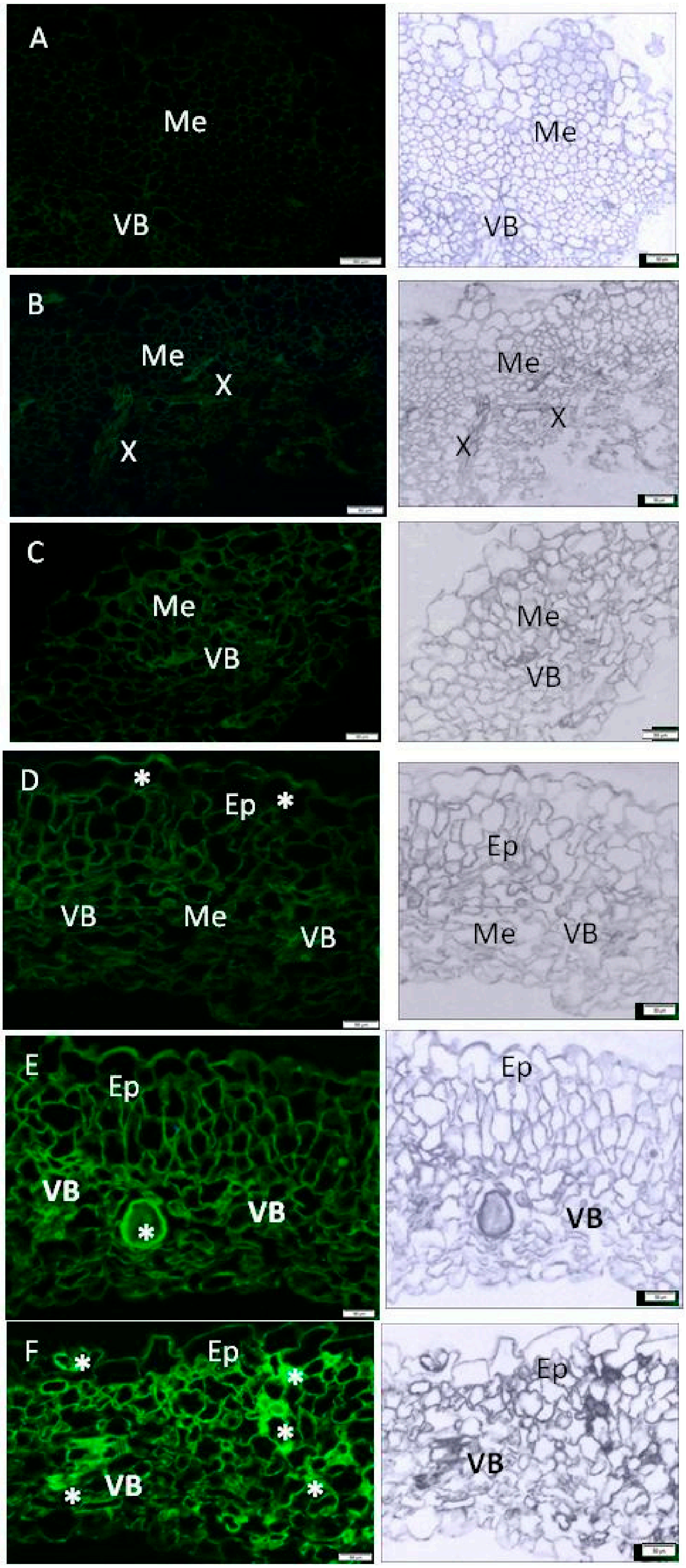

S1A Immunofluorescence localization of StEXPAs in PVY^{NTN} - potato Irys compatible (B-F) with corresponding light microscopy images. (A) StEXPAs green fluorescence signal in vascular bundle

and parenchyma cells in mock-inoculated Irys potato leaf. **(B)** StEXPAs green fluorescence signal in xylem tracheary elements and parenchyma cells in PVY^{NTN}-inoculated Irys potato leaf 0 day post inoculation **(C)** StEXPAs green fluorescence signal in vascular bundle and mesophyll cells 1 dpi **(D)** StEXPAs in external cell wall of epidermis (*), mesophyll cells and vascular tissues 7 dpi **(E)** StEXPAs in all leaf tissues14 dpi**,** the most intense signal in necrotizing mesophyll cell (*).**(F)** StEXPAs signal in all leaf tissues 30 dpi. The most intense signal (*) in cell wall and symplast in the area of necrotizing mesophyll cells, xylem tracheary elements and stomata. Bar 50µm, I-L Bar 20 µm. Ep-epidermis, Me-mesophyll, VB-vascular bundle, X-xylem tracheary elements .

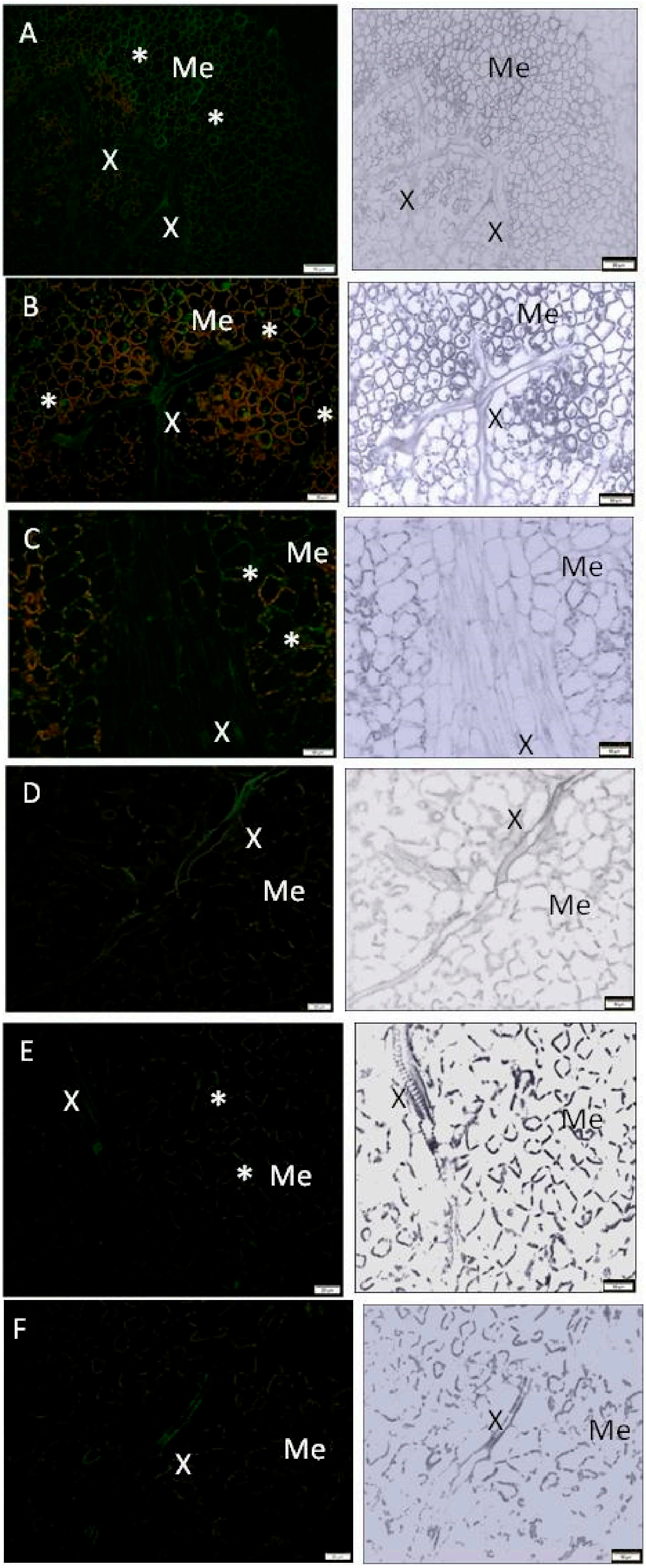
A
*
Me
*
X
X
Me
X
X
B
Me
*
*
X
*
Me
X
C
Me
*
*
X
Me
X
D
X
Me
X
Me
E
X
*
*
Me
X
Me
F
Me
X
X
Me

S1B Immunofluorescence localization of StEXPAs in PVY^{NTN} - potato Sárpo Mira incompatible interaction with corresponding light microscopy images. (A) Green fluorescence signal (*) in xylem tracheary elements and mesophyll cells in mock--inoculated Sárpo Mira potato leaf (**B**) StEXPAs green fluorescence signal (*) in xylem tracheary elements and mesophyll cells in PVY^{NTN}-inoculated Irys potato leaf 0 day post inoculation. (**C**) StEXPAs green fluorescence signal singular mesophyll cells 1 dpi. (**D**) StEXPAs green fluorescence signal (*) in xylem tracheary elements 7 dpi. (**E**) Weak signal of StEXPAs in singular mesophyll cells 14 dpi. **(F)** StEXPAs green fluorescence signal (*) in xylem tracheary elements 30 dpi. A-B Bar 50μm, C-F Bar 20 μm. Ep-epidermis, Me-mesophyll, VB-vascular bundle, X-xylem tracheary elements .

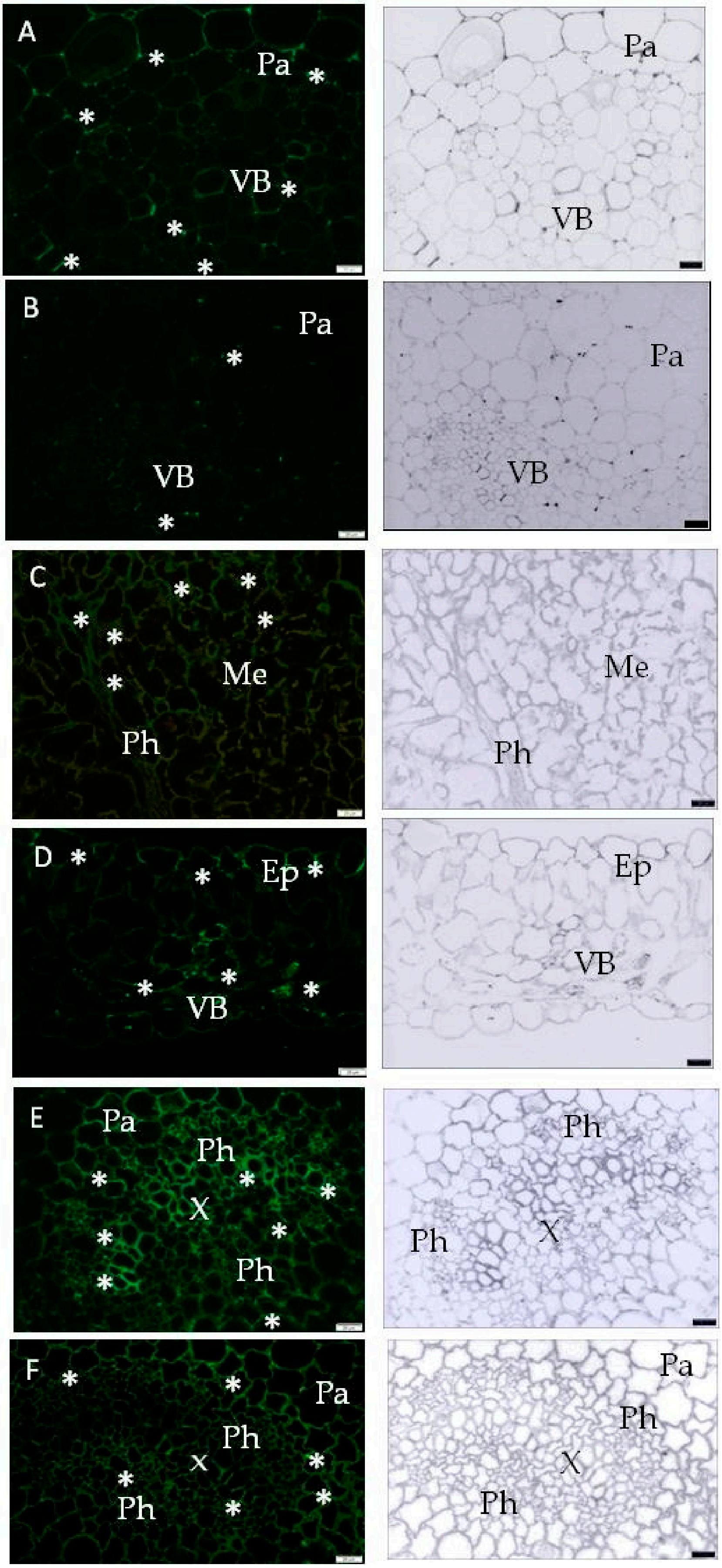
A
Pa
VB
Pa
VB
B
Pa
VB
Pa
VB
C
Me
Ph
Me
Ph
D
Ep
VB
Ep
VB
E
Pa
Ph
X
Ph
Ph
Ph
X
F
Pa
Ph
X
Ph
Pa
Ph
X
Ph

S1C Immunofluorescence localization of HRGPs-extensins in compatible PVY^{NTN}- interaction with corresponding light microscopy images. **(A)** HRGPs green fluorescence signal (*) in xylem tracheary elements and parenchyma cells in mock-inoculated Irys potato **(B)** HRGPs weak green fluorescence signal in parenchyma cell rounded vascular tissues 0 dpi **(C)** HRGPs in phloem and mesophyll cells 1dpi **(D)** Green fluorescence signal in epidermis and vascular bundle 7 days post virus inoculation **(E)** HRGPs in vascular bundle and parenchyma cells arounded vascular tissues 14dpi **(F)** HRGPs in vascular bundle and parenchyma 30 days post virus inoculation. Bar 20µm. Ep-epidermis, Me-mesophyll, Pa-parenchyma, Ph-phloem,VB-vascular bundle, X-xylem tracheary elements, XP-xylem parenchyma.

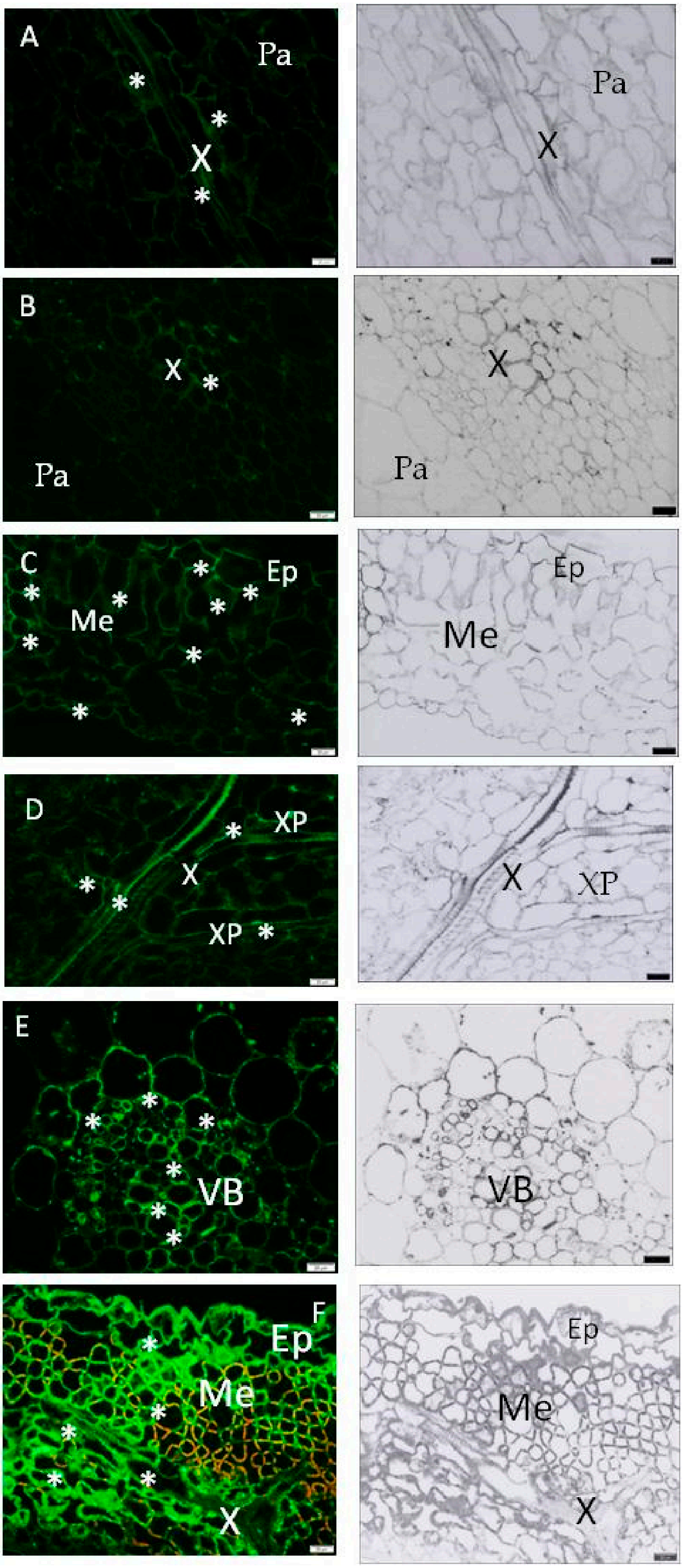

S1D Immunofluorescence localization of HRGPs-extensins in incompatible PVY^{NTN}- interaction with corresponding light microscopy images. (A) HRGPs green fluorescence signal (*) in xylem

tracheary elements in mock-inoculated Sárpo Mira potato **(B)** HRGPs green fluorescence signal in xylem tracheary elements in Sárpo Mira potato 0dpi **(C)** HRGPs in mesophyll and epidermis 1dpi **(D)** HRGPs in xylem tracheary elements and xylem parenchyma cells 7dpi **(E)** HRGPs in vascular bundle 14 dpi **(F)** HRGPs in epidermis, mesophyll and xylem tracheary elements 30 days post virus inoculation**.** Bar 20μm. Ep-epidermis, Me-mesophyll, Pa-parenchyma,VB-vascular bundle, X-xylem tracheary elements, XP-xylem parenchyma.
